# 6-Octadecenoic and Oleic Acid in Liquid Smoke Rice Husk Showed COVID-19 Inhibitor Properties

**DOI:** 10.1155/2024/8105595

**Published:** 2024-04-25

**Authors:** Ira Arundina, Neni Frimayanti, Meircurius Dwi Condro Surboyo, Theresia Indah Budhy, Benni Iskandar

**Affiliations:** ^1^Department of Oral Biology, Faculty of Dental Medicine, Universitas Airlangga, Surabaya 60132, Indonesia; ^2^Sekolah Tinggi Ilmu Farmasi (STIFAR), Pekanbaru, Riau, Indonesia; ^3^Department of Oral Medicine, Faculty of Dental Medicine, Universitas Airlangga, Surabaya 60132, Indonesia; ^4^Department of Oral Pathology and Maxillofacial, Faculty of Dental Medicine, Universitas Airlangga, Surabaya 60132, Indonesia; ^5^School of Pharmacy, College of Pharmacy, Taipei Medical University, Taipei, Taiwan

## Abstract

In recent years, liquid smoke rice husk (LSRH) has shown its therapeutic potency to diabetes, wound healing, stomatitis, and periodontitis. The phenol, 6-octadecenoic acid, oleic acid, and 9-octadecanoic acid were responsible for their therapeutic effect. The LSRH also demonstrated their potential for infectious diseases such as coronavirus disease (COVID-19). Therefore, the molecular dynamics (MDs) simulation and pharmacophore analysis was performed to analyse the binding stability of 6-octadecenoic and oleic acid. Based on MD simulation, 6-octadecenoic and oleic acids seemed to retain their interactions with Ser144 and Thr24, respectively, with hydrogen bond distance less than 2.9 Å. This interaction was stable during the simulation and has hydrophobic and hydrogen bonds/acceptors. The 6-octadecenoic acid and oleic acid were confirmed to have great potency as inhibitors for COVID-19. These compounds also showed that the existence of hydrophobic and hydrogen bonds/acceptors could increase biological activity.

## 1. Introduction

Liquid smoke has been long used by people in various parts of the world as a natural food preservative [1, 2], food flavouring [3], and pest repellent [4]. Over time, research on liquid smoke continued to grow, and even research on its efficacy and therapeutic potential has been observed. Various liquid smoke can be produced from wood through the pyrolysis process, one of which was the most being studied in Indonesia is coconut shell (*Cocos nucifera* L) [5], rice husk (*Oryza sativa*) [6, 7], durian peel [8], corn comb [9], cocoa bean skin [10, 11], cashew nut shell [12], palm shell [13], Medang wood (*Cinnamomum* sp.) [14], and cajuput (*Melaleuca leucadendron*) [15].

One of the various liquid smokes that has been widely researched was liquid smoke from rice husk (LSRH). The potential for therapy has also been widely studied, including as a therapy for diabetes [16–18], wounds [19], canker sores [20], and periodontitis [7], until the latest one was for COVID-19 [21]. Liquid smoke also had various properties such as antibacterial [22, 23], antifungal, antioxidant [11, 24], analgesic, and anti-inflammatory [25]. These various properties and capabilities were due to the fact that liquid smoke had different complex contents, including phenol, mequinol, guaiacol, 6-octadecenoic acid, oleic acid, and 9-octadecanoic acid [26]. The LSRH has previously been confirmed to have potential as a COVID-19 protease inhibitor through a phenol, mequinol, 2-methoxy-phenol, 6-octadecenoic acid, oleic acid, and 9-octadecenoic acid [21]. Only the 6-octadecenoic acid and oleic acid had the binding-free energy and factor of binding presumably as active COVID-19 inhibitors [21].

One strategy that can be used to analyse an active compound from a natural product that can work precisely to target protein was through an in-silico study. This study provides a prediction interaction between active compounds to target protein through docking molecular and its stability analysis using MD [27, 28]. Based on that reason, the MD and pharmacophores analysis was carried out to confirm the binding stability of 6-octadecenoic acid and oleic acid from LSRH to COVID-19.

## 2. Materials and Methods

### 2.1. Compound Material

The molecular structure of the compounds, i.e., 6-octadecenoic acid and oleic acid, was sketched using Chemdraw Professional 15.0 and saved in “.cdx” format. The 3D structure was prepared using the Molecular Operating Environment (MOE) program 2020.0901 with MMFF94x force field and 0.0001 gradients. Then, it was saved in “mdb” format. Based on the docking results from our previous research these two compounds (i.e., 6-octadecenoic acid and oleic acid), complexes with protein were selected to perform MD simulation [21].

### 2.2. Molecular Dynamic

MD simulation was generated using complexes of ligands (i.e., 6-Octadecenoic acid and oleic acid) with protein main protease (M^pro^). This protein was taken from the protein database with PDB ID 6LU7 with a resolution of 2.6 Å. The MD simulation was carried out using the NAMD (NAnoscale Molecular Dynamics software) version 2.9. (i.e., preliminary study). The best force field of CHARMM27 (Chemistry at HARvard Macromolecular Mechanics) was selected as the best force field. This force field was used to perform MD simulations for each tested compound. A TIP3P water box with a 2.5 water layer for each direction of the coordinated structure was employed to mimic the protein [29, 30]. To replicate physiological conditions, a salt solution of 0.15 M NaCl was introduced. The molecular dynamics (MDs) simulation was conducted under conditions ensuring thermodynamic stability.

The apparatus was gradually heated by an NVT ensemble applied over 100 ps from 0 to 300 K. Each system in an isothermal, isobaric ensemble (NPT) with periodic boundary conditions underwent MD simulations on a time scale of 50 ns. Temperature and pressure parameters were coupled at a rate of one ps. When sampling, the coordinates were recorded every 0.1 ps. For further binding-free energy calculations and the breakdown process, the conformations generated by the simulations were employed.

Followed by heating and equilibration, these systems underwent a production MD run in an NPT ensemble for 50 ns [31–34]. The software produced a 2D graph showing approximately inherent dynamical stability as determined by the root mean square deviation (RMSD) and root-mean-square fluctuation (RMSF).

### 2.3. Pharmacophore

The energy of each molecular structure was minimised using the MMFF94 × force field to a gradient of 0.00001 kcal/mol/Å. Descriptors of those compounds were generated, and at the same time, the pharmacophore of the ligands was performed. In this study, the best alignments of pharmacophores for 6-octadecenoic acid were generated using three features, hydrogen bond donor, hydrogen bond acceptor, and hydrophobic properties. Oleic acid was developed with two features, hydrogen bond donors, and acceptors. These pharmacophore features were then used to confirm that the model can be used for predicting the biological activity of these compounds. Pharmacophore was performed using MOE 2021.0901 (chemical-computing group) software package.

## 3. Results

### 3.1. Molecular Dynamic

Previous research has reported that one of the main proteases (M^pro^) is 6LU7. It can be used as a protein target for SARS-CoV-2; thus, in this research, we used this protein as the main target for predicting whether these ligands have the potential to be used as COVID-19 inhibitors.

**Figure 1:**
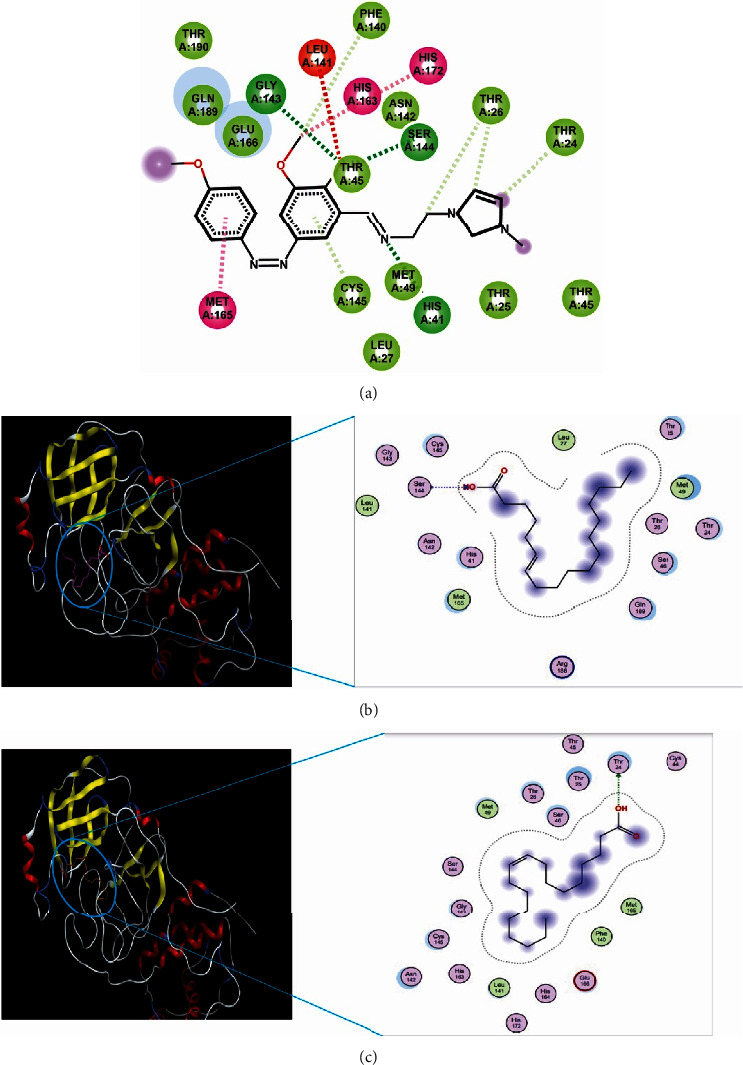
Visualisation of MD simulation for protein 6LU and native ligand (a), 6-octadecenoic acid (b), and oleic acid (c). Van der Waals interaction (bold red cycle); hydrophobic interaction (bold blue cycle); hydrogen bond interaction (green or blue dash line); an amino acid that involves in binding site through other interaction (other colours).

MD simulation was performed on protein 6LU7 and native ligand (N-[(5-methylisoxazol-3-yl)carbonyl]alanyl-l-valyl-N∼1∼-((1R,2Z)-4-(benzyloxy)-4-oxo-1-{[(3R)-2-oxopyrrolidin-3-yl]methyl}but-2-enyl)-l-leucinamide [35]. It was shown that this protein interacts with Thr24 and Thr26 through hydrogen bond interaction. In addition, this compound also has Van der Waals interaction with Leu41. It has RMSD of 0.28 nm. MD of the complex of native ligand protein allowed for validation to determine the degree of affinity prediction strength.  Figure 1(a) shows the results of the validation. It demonstrated that the natural ligand's RMSD value was 0.28 nm and that a binding energy of −29.186 kcal/mol was achieved.

Molecular docking has been successfully performed before constructed MD simulation [21]. Based on MD simulation, the 6-octadecenoic interacts with Ser144 and oleic acid interacts with Thr24, before and after simulation. On the other hand, the 2-methoxy-phenol only interacted with Asn142 before simulation and lost the interaction during MD simulation. Visualisation of MD simulation for 6-octadecenoic acid and oleic acid are depicted in Figures 1(b) and 1(c).

The interaction of 6-octadecenoic and oleic acid were also presented with hydrogen bond distance, with a capability of less than 2.9 Å. It was indicated that 6-octadecenoic acid and oleic acid were stable with variations in pressure or temperature (Table 1).

The root-mean-square deviation (RMSD) and root-mean-square fluctuation (RMSF) examined the complex dynamic behaviour. The conformational stability of a complex, structural, and dynamic measure was evaluated using the RMSD. A protein was less stable if its RMSD value were higher. Based on this calculation, complex 6-octadecenoic acid protein seemed to have oscillations 40 ns with an average RMSD of 0.32 nm. Prior to 10 ns, there was some variance in the average RMSD of the oleic acid-protein complex, but it was still constant for the simulation time. This complex had an average RMSD of roughly 0.32 nm. Both complexes were stable and have quite strong bonding as evidenced by the reduced average value and volatility of the RMSD in both complexes, which are nearly identical. A lower RMSD value indicated greater stability of the compound.

**Figure 2:**
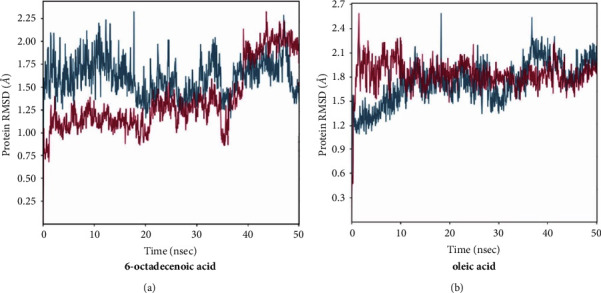
(a) RMSD M^pro^-protein (blue) and 6-octadecenoic acid-protein (pink) and (b) RMSD M^pro^-protein (blue) and oleic acid-protein (pink).

The RMSD values of both complexes, namely, M^pro^ and 6-octadenoic acid indicated structurally stable conformations throughout all simulations, hovering around RMSD values of approximately 2.0–2.4 Å and 2.5 Å, respectively, relative to the simulation's starting point. There is no significant difference observed in the RMSD values of compounds 6-octadecenoic acid and compound oleic acid when in complex with the M^pro^ enzyme of SARS-CoV. The RMSD oscillates within a range of less than 2.25 Å–2.8 Å in the protein complex with oleic acid at the 50 ns mark. Conversely, for the protease of SARS-CoV-2, 6-octadenoic acid exhibits RMSD values lower than 2.4 Å, falling within an acceptable range (<4 Å). The RMSD for these compounds is depicted in Figure 2.

The alteration of a lead molecule to increase activity can be guided by the examination of hydrogen bonds, which can provide important insights into the stability of a ligand-protein complex. The angles and spacings of the donor, hydrogen, and acceptor atoms can be used to calculate the strength of the hydrogen bond. Greater distances and smaller angles suggest a weaker link, but smaller distances and angles of approximately 180° indicate a strong bond [36]. In our case, the angle is almost approximately 180°. It is indicated that hydrogen bonding is strong.

**Figure 3:**
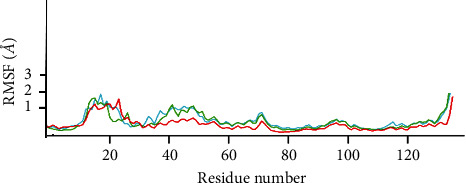
RMSF of 6-octadecenoic acid, oleic acid, and amino acid residue.

RMSF was used to monitor the fluctuation of amino acid residue [37]. The RMSF graph versus amino acid residue number was depicted in Figure 3. It also demonstrated that there were many variations of amino acids involved during the MD simulation. The stability of the compound was shown with survival of the amino acid fluctuation being under 4 Å. The amino acids of Thr24 and Ser144 showed high variability and stability for 6-octadecenoic acid and oleic acid.

Based on RMSD, RMSF, and hydrogen bonding results from MD simulation, it was indicated that 6-octadecenoic acid and oleic acid were binding well with Ser144 and Thr24, respectively.

The average binding energy for 6-octadecenoic acid and oleic acid-protein complexes was calculated from 2000 frames using the MMPBSA method. The bootstrap module of MMPBSA with APBS was utilized to determine the average binding energy along with the standard deviation or standard error. The results revealed the following average binding-free energies for the respective complexes: −28.987 ± 0.133 kJ/mol (6-octadecenoic acid) and −17.064 ± 0.376 kJ/mol (oleic acid). The evaluation of binding-free energy indicates that 6-octadecenoic acid exhibited significantly more negative binding energy than oleic acid. The effectiveness of ligands against the template target M^pro^ is determined by the stable interactions formed between the ligands and the binding site of the mentioned amino acids. The binding-free energy of these ligands during MD simulation is depicted in Table 2.

### 3.2. Pharmacophore

**Figure 4:**
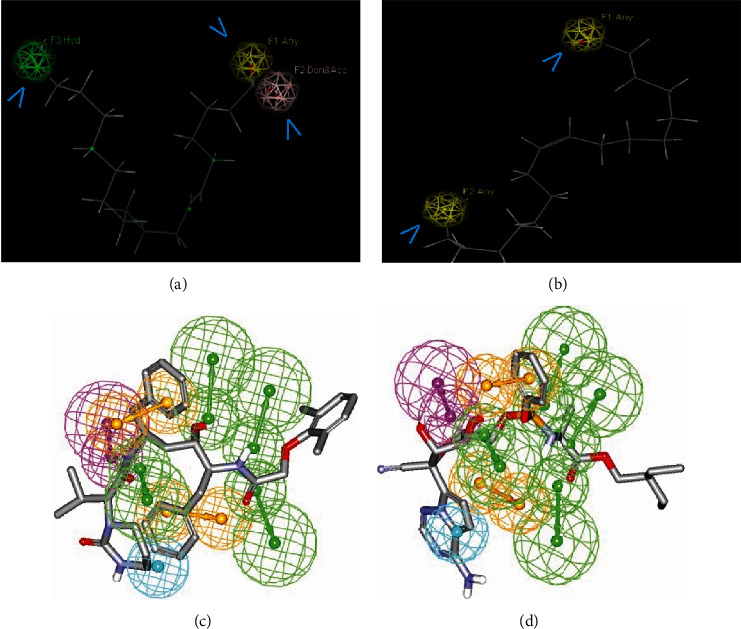
The best pharmacophore hypothesis for 6-octadecenoic acid and oleic acid. Pharmacophores were hydrogen bond donors/acceptors (colour coded with yellow/pink) and hydrophobic bonds (coloured green). (a) 6-octadenoic acid, (b) oleic acid, (c) lopinavir, and (d) remdesvir.

The interaction of 6-octadecenoic acid and oleic acid were shown by pharmacophore simulation presented in Figures 4(a) and 4(b). The 6-octadecenoic acid had a hydrophobic sphere (green) and a hydrogen bond donor/acceptor. On the other hand, oleic acid had only a hydrogen bond donor/acceptor. The hydrophobic sphere (green) and hydrogen bond donor/acceptor (yellow/pink) generate the best pharmacophores hypothesis. These pharmacophores were considered the key elements; they contribute to ligand activity.

As reference, pharmacophore for some of potential inhibitor for COVID-19 (i.e., lopinavir and remdesivir) was also done to validate the pharmacophore of 6-octadenoic acid and oleic acid (Figures 4(c) and 4(d)). The hydrophobic sphere (green) and hydrogen bond donor/acceptor (yellow/pink) generate the best pharmacophores hypothesis [38]. It is similar with the new ligands, i.e., 6-octadenoic acid and oleic acid.

## 4. Discussion

The main protease of SARS-CoV-2, also recognized as 3C-like protease (3CLpro) or main protease (M^pro^), is a crucial enzyme in the virus's replication, responsible for processing viral polyproteins translated from viral RNA. This enzyme cleaves polyproteins at specific sites, releasing individual functional viral proteins essential for assembling new virus particles. The SARS-CoV-2 main protease is a potential target for antiviral drug development since inhibiting its activity could disrupt viral replication [39]. Researchers actively explore strategies like structure-based drug design to identify small molecules inhibiting the main protease, potentially serving as antiviral drugs. Understanding the main protease's structure and function has been pivotal in developing therapeutic interventions against COVID-19. The three-dimensional structure of SARS-CoV-2's main protease has been determined, offering valuable insights for drug design. This information helps researchers identify compounds specifically targeting and inhibiting the main protease, disrupting the viral life cycle [40].

LSRH was proposed as a candidate for a drug, one of which was for COVID-19, with targeted to M^pro^. This proposal was not only based on assumptions but on various data and research that has been done previously. LSRH contains various components although its acidic nature predominates [16]. The main component of LSRH was reported differently in various studies, with percentages varying as follows: 2-methoxyphenol (13.45%), mequinol (13.45%), phenol (10.52%), 6-octadecenoic acid (7.81%), oleic acid (7.81%), 9-octadecenoic acid (7.81%), and 2-methoxy-5-methylphenol (4.88%) [41]. Another study also mentioned that the LSRH contained 116 components dominated by phenol (10.99%) and 2-methoxy phenol (3.75%) but did not contain 6-octadecenoic acid, oleic acid, and 9-octadecenoic acid [16].

The LSRH possessed antidiabetic activity which was shown by controlling fasting blood glucose, maintaining the lipid level, preventing pancreas cell destruction, and producing radical and various proinflammatory cytokines [16–18]. The antibacterial activity inhibited the *Porphyromonas gingivalis* as an aetiology of periodontitis [7], stimulated the proliferation of osteoblast [42], inhibited the interleukin‐1® (IL‐1®), and stimulated nuclear factor erythroid‐2 (Nrf‐2) [43]. In mucosal disease, the LSRH stimulated various growth factors such as fibroblast growth factor (FGF), vascular endothelial growth factor (VEGF), and platelet-derived growth factor (PDGF), transforming growth factor (TGF-*β*) [20]. At the cellular level, LSRH showed effects on recruiting and proliferation of macrophages, lymphocytes, fibroblasts, and collagen type 1 [20].

The LSRH had previously been confirmed to have potential as a COVID-19 protease inhibitor through a phenol, mequinol, 2-methoxy-phenol, 6-octadecenoic acid, oleic acid, and 9-octadecenoic acid. Only two components, 6-octadecenoic acid and oleic acid, had the binding-free energy and factor of binding presumably as active COVID-19 inhibitors [21]. The two-component (i.e., 6-octadecenoic acid and oleic acid) form LSRH was chosen as an active component based on the previous research and its ability to interact with the main protease of coronavirus [21]. These compounds were subjected to MD simulation to investigate the interactions between ligands and receptors [44]. To corroborate the binding profile of the ligands and to provide a general sense of the estimated active compounds, the stability of the MD simulation was investigated. The simulation was allowed to continue for 50 ns [31–34]. To make sure that the connection between protein and active chemicals was still maintained in the current investigation, an MD simulation was carried out [45]. To check the ligand's affinity to the binding site, it was started by utilising high stability with an energy minimum of 300 K.

The effectiveness of hydrogen binding in the potentially active compounds, 6-octadecenoic and oleic acid, were evaluated before and after a 50 ns and 300 K MD simulation to check the results. The spatial arrangement shows that these compounds bind well with identical residues before and after the operation. In addition, the hydrogen bond distance was less than 2.9 Å with the hydrogen bond angles as 155° and 160°. The distance less than and 2.9 Å angle criteria 150–180° for hydrogen bond interactions are key factors that define the strength and specificity of these interactions. It was indicated that 6-octadecenoic acid and oleic acid were stable with variation in pressure or temperature and these compounds also have big potentiality as active compounds against COVID-19. The other compounds, such as phenol, mequinol, and 9-octadecenoic acid, seemed to lose their activity due to the interaction between ligand and receptor that was not maintained. Based on MD simulation, these compounds could not maintain the existence of hydrogen bonds and the distance of hydrogen bonding of 2.9 Å [46]. In the present study, even though three of these compounds did not have hydrogen bonding, another interaction was broken after the MD simulation. It made all three of these compounds inactive.

In addition, other parameters for MD simulation are RMSD, RMSF, and free-binding energy. The RMSD has traditionally served as a metric for gauging the separation between the atomic chains of proteins, assessing disparities in two protein structures over simulation intervals. A higher RMSD signifies greater dissimilarity, while a value of zero denotes an identical conformational structure. Based on Figure 2, it seems that 6-octadecenoic acid and oleic acid were stable because the RMSD value is not so high. The RMSF is employed to evaluate the flexibility of individual residues and quantify their movement or fluctuation throughout a simulation duration. In practical terms, it identifies the amino acids within the protein sequence that contribute more significantly to structural motion. Based on Figure 1, it showed that there are two amino acids, i.e., Thr24 and Ser144 that bonded with 6-octadecenoic acid and oleic acid, thus it exhibited considerable variability and stability [47]. Regarding the free binding energy, it is shown that the 6-octadecanoic acid has stable binding to target closely relative similar to the native ligand.

Pharmacophore models were described as the steric and electronic properties required to enable the best supramolecular interactions with a particular biological target structure and initiate (or inhibit) that structure's biological response. H-bond acceptors (HBAs), H-bond donors (HBDs), positive and negative ionizable groups (PI/NI), hydrophobic regions (H), and aromatic rings (ARs) are some of these characteristics. A set of crucial pharmacophore properties that characterise the ligand's binding mode were extracted from a protein-ligand complex's 3D structure using structure-based pharmacophore models. These pharmacophore models can then be employed with virtual screening techniques to find new active chemicals [48, 49].

This study investigated the possibility of using pharmacophore models through MD simulations. Preliminary results were presented, which extend the analysis conducted for one protein-ligand system. The investigation studied the pharmacophore model's variability and analysed the occurrence of features as a function of time. From this analysis, two pharmacophore models were derived based on the frequency of interactions and the time-resolved dynamics of the pharmacophore features.

The hydrophobic sphere and hydrogen bond donor/acceptor generated the best pharmacophores hypothesis. These pharmacophores were considered the key elements since they contributed to ligand activity [50, 51]. The pharmacophore hypothesis for 6-octadecenoic acid and oleic acid was archived. It showed the importance of the hydrophobic and hydrogen bond donor/acceptor features that can enhance biological activity [52]. The result of this study strengthened the LSRH, potentially COVID-19, because of the presence of 6-octadecenoic and oleic acid.

Two of the COVID-19 inhibitors was performed for their pharmacophore. The constructed pharmacophore includes the following features: hydrogen bond acceptors (HBAs), hydrogen bond donor (HBD), aromatic rings (RingArom), and hydrophobic feature (Hbic). It is commonly known that the selectivity of a pharmacophore is strongly influenced by its feature count; complex pharmacophores with a high feature count are more selective than simple ones with a low feature count. These characteristics of the pharmacophore produced here will improve its selectivity, limit the number of hits that are recorded, and raise the likelihood that actual positive hits are appropriately captured. It was used as the reference for pharmacophore validation of unknown compounds [38].

This was the first study to analyse the potential therapeutic of LSRH on COVID-19. With findings of this study, it was possible to expand the economic value of LSRH with its therapeutic potential for COVID-19. Not only for periodontitis and ulcer, but the LSRH also showed its therapeutic potential for COVID-19. Further studies are needed to confirm the possible mechanism.

## 5. Conclusions

Based on MD simulation, it was confirmed that two active compounds, i.e., 6-octadecenoic acid and oleic acid, were confirmed to have great potency as inhibitors for COVID-19. These compounds retain their interactions with Ser144 and Thr24, respectively, with stable hydrogen bond interaction. This is an early stage for discovering potential compounds for COVID-19 inhibitors. However, the *in vitro* and *in vivo* evaluations in further work are required to confirm the potencies of these 6-octadecenoic acid and oleic acid.

## Figures and Tables

**Table 1:** The molecular binding and molecular interaction during MD simulation.

Compound	After docking	MD simulation	Distance of hydrogen bond interaction	Angles of hydrogen bond interaction
Phenol	—	—	—	
Mequinol	—	—	—	
2-Methoxy-phenol	Asn142	—	—	
6-Octadecenoic acid	Ser144	Ser144	2.9 Å	160°
9-Octadecenoic acid	—	—	—	—
Oleic acid	Thr24	Thr24	2.9 Å	155°
Native ligand	Thr24, Thr26	Thr24, Thr26	2.9 Å	170°

^*∗*^MD = molecular dynamic.

**Table 2:** The binding-free energy for the tested compound.

No	Compound	Binding free energy (kJ/mol)
1	6-Octadenoic acid	−28.987
2	Oleic acid	−17.064
3	Native ligand	−29.186

## Data Availability

The data used to support the findings of this study are available from the corresponding author upon request.
